# Automated counting of prostate cell types with image processing and machine learning

**DOI:** 10.1371/journal.pone.0340628

**Published:** 2026-05-04

**Authors:** Babak Kamali Doust Azad, Aryan Norouzzadeh Hakimi, Seyed Mohammad Tabatabaei, Bahar Mohammadi Arezouchi, Mohsen Mashhadi Keshtiban, Akram Mirzaei, Fatemeh Nafian, Fatemeh Khatami, Mohammad Kazem Aghamir, Mohammadreza Kolahdouz

**Affiliations:** 1 School of Electrical and Computer Engineering, College of Engineering, University of Tehran, Tehran, Iran; 2 Department of Biomedical Engineering, Tehran Islamic Azad University of Medical Sciences, Tehran, Iran; 3 Department of Mechanical Engineering, Tarbiat Modares University, Tehran, Iran; 4 Urology Research Center, Tehran University of Medical Sciences, Tehran, Iran; 5 Department of Medical Laboratory Sciences, TeMS.C., Islamic Azad University Tehran, Tehran, Iran; Bayer Crop Science United States: Bayer CropScience LP, UNITED STATES OF AMERICA

## Abstract

Traditional cell counting in clinical and research settings often relies on hemocytometry, a manual technique that is labor-intensive and prone to human error. These limitations in precision and throughput can hinder the development of effective diagnostic and therapeutic strategies, particularly in the context of prostate cancer. Recent advances in machine learning have shown considerable promise in enhancing the accuracy and efficiency of cell enumeration. In this study, we present a novel software system for the automated counting of prostate cancer cells, integrating image processing with deep learning methodologies. Unique to our approach, the system robustly utilizes images acquired from conventional mobile phone cameras, offering a highly accessible and scalable solution. It applies a convolutional neural network (CNN) in conjunction with a selective search algorithm to accurately identify regions of interest (ROIs), followed by robust image analysis algorithms for precise cell detection and quantification. This two-stage pipeline addresses the inherent variability and extraneous content in mobile-captured images, which is a significant advancement over methods reliant on controlled microscopic environments. Experimental evaluations demonstrate that the proposed method achieves superior accuracy compared to conventional manual counting approaches. This automated framework offers a practical, scalable solution that may significantly improve the reliability and efficiency of cell counting in both research and clinical diagnostics.

## Introduction

Prostate cancer (PCa) is the second most commonly diagnosed malignancy in men and the fifth leading cause of cancer-related mortality worldwide, with an estimated 400,000 deaths annually. Due to its asymptomatic nature in early stages, it often requires active clinical surveillance for timely intervention [1]. In prostate cancer research, three cell lines—LN-CaP, PC3, and DU145—are predominantly utilized to model different aspects of disease progression and treatment response [2]. LN-CaP cells, which are androgen-dependent, serve as a valuable model for studying early-stage, hormone-sensitive prostate cancer and for assessing the efficacy of hormonal therapies [3]. In contrast, PC3 cells, which originate from bone metastases and are androgen-independent, are commonly used to investigate aggressive and metastatic forms of the disease [4]. DU145 cells, recognized for their rapid proliferation, are employed in studies exploring tumor growth dynamics and metastatic behavior [5].

Precise cell enumeration is essential for a wide range of experimental procedures and therapeutic evaluations. Applications such as routine cell culture maintenance, quantitative PCR, xenograft modeling, and cell therapy all require accurate cell counts to ensure experimental consistency and reproducibility [6]. The hemocytometer remains the conventional tool for cell counting, enabling manual quantification through chambers etched with grid patterns. Although it provides a standardized approach, this method is labor-intensive and inherently susceptible to observer variability [7]. Accurate cell quantification is crucial for ensuring reliable experimental setups, assessing treatment outcomes, and monitoring cellular behavior under varying conditions [8]. Inaccurate cell counts can compromise the validity of experimental findings and hinder the development of effective therapeutic strategies. Therefore, reliable and reproducible cell enumeration forms the foundation of both research and clinical decision-making [9].

Despite its longstanding use, manual hemocytometry is time-consuming and vulnerable to human error. It requires the operator to visually count cells across chamber grids—an approach that is inefficient and not scalable for high-throughput studies [10]. To overcome these limitations, automated methods using image processing have been developed. Early approaches involved the use of hand-crafted features to distinguish cells based on geometric attributes such as shape and size [11]. These techniques, however, often require extensive preprocessing of microscopic images, especially when the images contain non-uniform regions unsuitable for analysis. Preparing such images for analysis is time-consuming and often necessitates manual cropping or adjustment, delaying processing workflows.

The integration of image processing in cellular analysis has introduced numerous benefits, including enhanced speed, improved accuracy, and automation. These advantages are particularly valuable in cancer diagnostics and biomedical research, where high-throughput and reliable analysis are critical [12–14]. Several algorithms have been proposed for automated cell detection and counting, including watershed segmentation, thresholding, and machine learning-based methods [15]. While each algorithm presents specific strengths and limitations, many of them still require well-prepared input images and struggle with accuracy when processing complex or noisy data [16,17].

Recent advances in deep learning have significantly improved cell counting capabilities. Deep learning algorithms, with their ability to extract high-level features from raw images, have shown excellent performance in detecting and classifying cells with minimal preprocessing [18,19]. These models are particularly useful in large-scale studies and clinical applications, where both accuracy and efficiency are paramount.

In light of these developments, this study aims to develop an automated system for counting prostate cancer cell lines (PC3, LN-CaP, and DU145) using a two-stage pipeline involving image processing and deep learning. The first stage focuses on the automated extraction of the region of interest (ROI) using images captured via mobile phone cameras. The second stage applies advanced image processing and machine learning techniques to accurately detect and enumerate the cells within the selected ROI. The performance of the system is evaluated to assess its potential as a practical tool for laboratory and clinical applications. Crucially, this work distinguishes itself by developing a robust solution specifically tailored for images captured by common mobile phone cameras, which often present challenges such as varying illumination, non-uniform backgrounds, and the presence of extraneous non-ROI regions, unlike controlled microscope environments. Our novel contribution lies in the synergistic combination of a deep Convolutional Neural Network (CNN) for accurate ROI identification and alignment with a selective search algorithm, followed by a refined sequence of classical image processing techniques (morphological operations, Canny edge detection, and Hough Circle Transform) for precise cell enumeration. This integrated pipeline is designed to overcome the limitations of manual hemocytometry by providing a user-friendly, cost-efficient, and highly accurate automated alternative that is accessible in diverse clinical and research settings.

## Methodology

**Fig 1:**
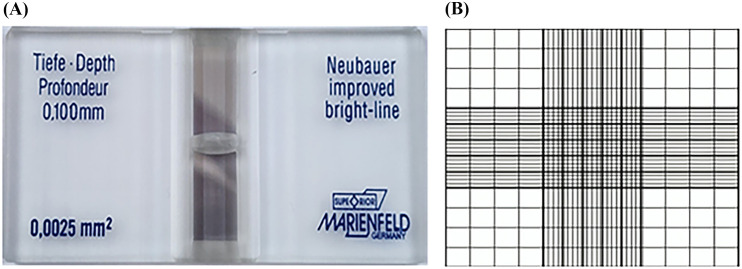
A) A hemocytometer – B) Hemocytometer segmentations.

Conventional cell counting is typically performed using a hemocytometer, a precision instrument consisting of four chambers etched with a grid pattern and overlaid by a coverslip (Fig 1). Each chamber contains a defined grid of 1 mm^2^, corresponding to a volume of 0.1 mm^3^. To estimate cell concentration, the operator manually counts the number of cells within the grid and calculates the average cell density based on the known chamber volume.

In the present study, the general procedure for cell counting was automated through the development of a software platform capable of processing images captured by mobile phone or camera. The system employs a convolutional neural network (CNN) in combination with a selective search algorithm to identify and extract the region of interest (ROI) from the input image. Following ROI extraction, image processing techniques are applied to detect and enumerate the cells. Additionally, a web-based interface was developed to provide users with an accessible online platform for performing automated cell counting.

## ROI Extraction

Accurate cell counting requires the precise identification of the region of interest (ROI) within each input image. This step is essential, particularly because images are captured using mobile devices, which may include extraneous areas that are not suitable for analysis. To automate ROI detection, a combination of a Convolutional Neural Network (CNN) and a selective search algorithm was employed to distinguish between ROI and non-ROI regions.

CNNs are widely used in image classification, similarity-based image clustering (e.g., photo search), and object recognition in complex scenes. These networks are capable of recognizing diverse visual patterns such as facial features, tumors, and other structural components within image data [20]. The fundamental component of a CNN is the convolutional layer, which processes input data through a set of learnable filters or kernels. CNNs also incorporate fully connected layers that perform classification by assigning categorical labels to the extracted features [21].

To effectively train the CNN model, a diverse dataset of hemocytometer images was curated, containing examples from both ROI and non-ROI regions. These images were labeled into two binary classes using an encoder-based approach. Data augmentation techniques were applied to improve generalization and reduce overfitting. Prior to training, all images were resized to 256 × 256 pixels—a common preprocessing step in CNN workflows that reduces computational complexity while preserving critical features [22]. Specifically, as implemented in our training code, images from ‘Non_Roi’ and ‘ROI’ directories were collected, resized to 256x256 pixels, and labeled using `OneHotEncoder` for binary classification (0 for Non-ROI, 1 for ROI). The dataset was then split into 90% for training and 10% for testing. Data augmentation, specifically `horizontal_flip = True` using `ImageDataGenerator`, was applied to the training data to enhance the model’s robustness and prevent overfitting.

**Fig 2:**
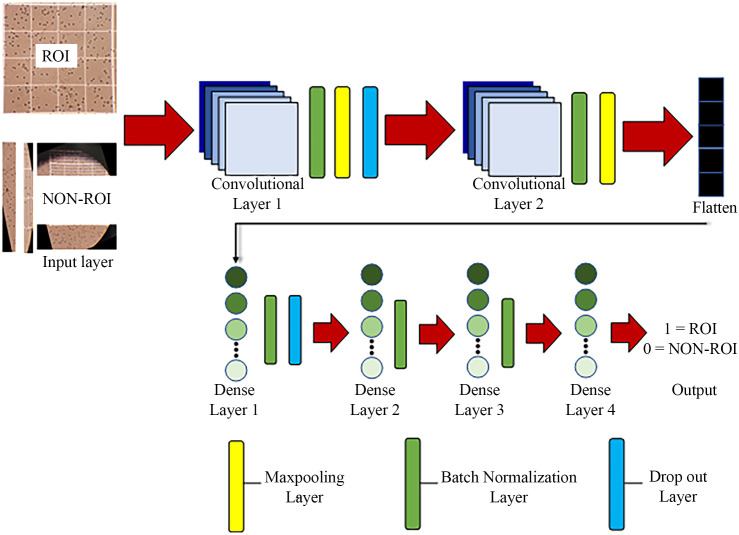
Training processes of the CNN and ANN model used for identifying the correct ROI image.

The CNN architecture used in this study comprises two convolutional layers and two max-pooling layers. The first convolutional layer utilizes a 2 × 2 kernel repeated 256 times, followed by a second convolutional layer with the same kernel repeated 128 times. Two BatchNormalization layers were added to stabilize learning by normalizing feature distributions. A dropout layer was included to mitigate overfitting. The output features extracted by the CNN are then passed to an Artificial Neural Network (ANN) for classification. The ANN model consists of four fully connected (dense) layers, with 128 neurons in the first two layers, 64 neurons in the third, and 2 neurons in the final output layer. The ANN also incorporates two BatchNormalization layers and one dropout layer. The training process is illustrated in Fig 2. This relatively shallow architecture was chosen after empirical evaluation to optimize between performance and computational complexity for the specific task of ROI/Non-ROI classification from mobile phone images. While deeper architectures exist, this design proved highly effective in accurately identifying the hemocytometer region while being efficient for deployment, achieving strong convergence in training and validation accuracy as shown in Fig 6. The model was compiled with ‘categorical_crossentropy’ loss and the ‘adam’ optimizer, and trained for 10 epochs with a batch size of 32.

**Fig 3:**
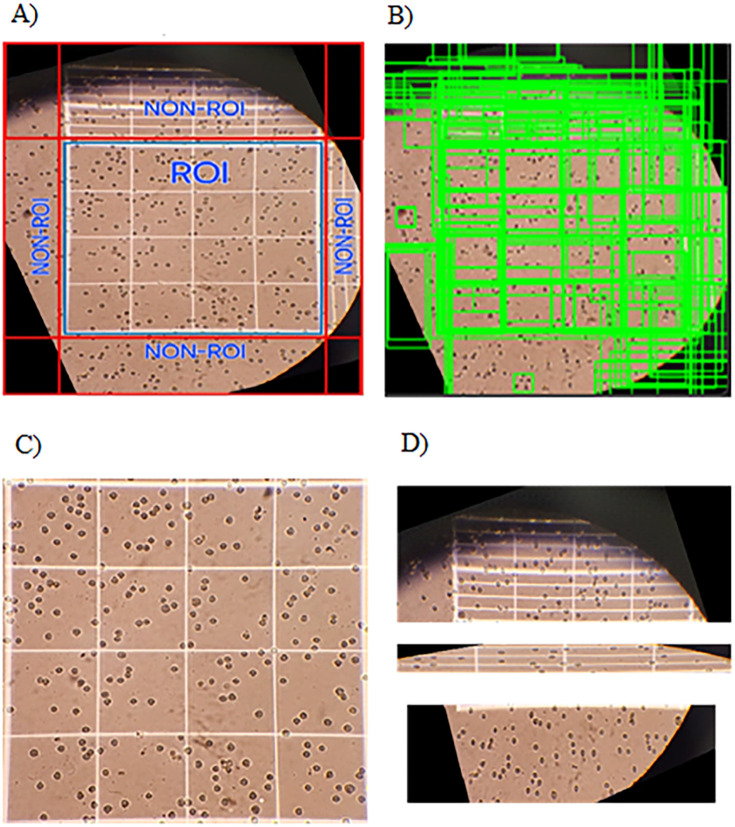
Selective Region Proposal and ROI Cropping in Automated Prostate Cell Analysis. **(A)** A sample image taken from a mobile phone; **(B)** extracted regions after running selective search algorithm; **(C)** desirable cropped ROI, **(D)** undesirable cropped image.

**Fig 4:**
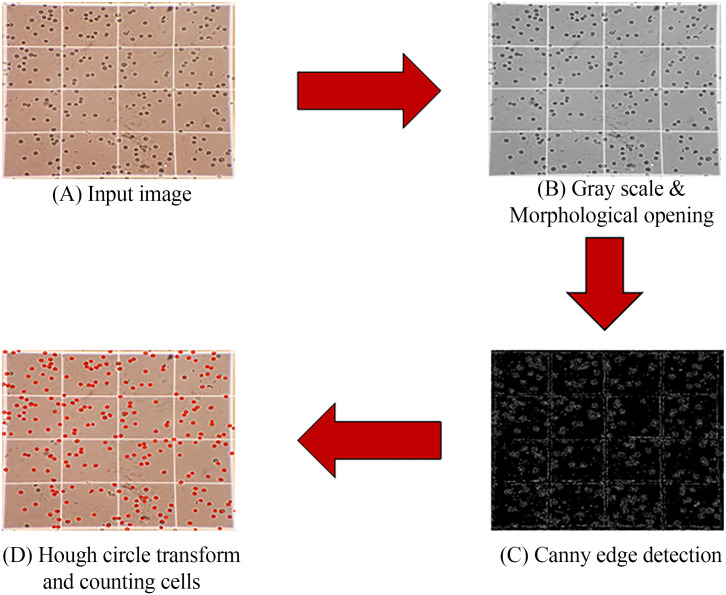
Image processing steps including (A) The Input image, (B) Morphological opening operator on a gray scale image, (C) Canny edge detection, (D) Hough circle transform and counting cells.

**Fig 5:**
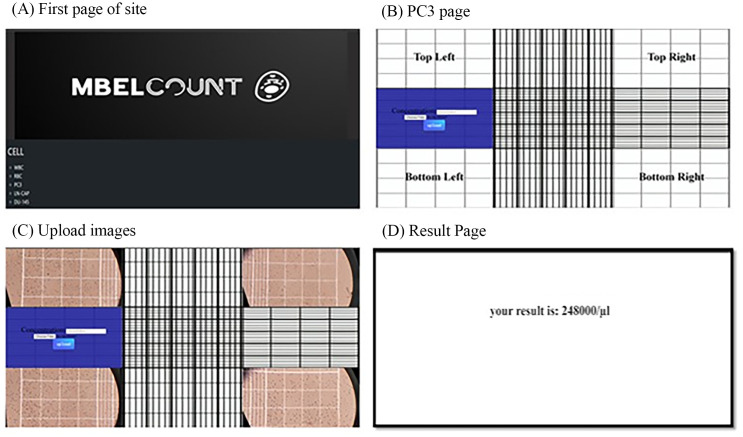
Web-based workflow for automated counting of cell types. **(A)** The first page of the website for selecting the cell type, **(B)** uploading images for each chamber, **(C)** uploaded images for each chamber, **(D)** the cell count is displayed on the screen.

While CNNs are effective for global semantic understanding of images, they may not focus specifically on smaller regions containing cells. To address this, a selective search algorithm was integrated into the pipeline to assist in ROI localization. This algorithm segments each image into numerous small rectangular regions and generates a hierarchical grouping, ranking regions based on their likelihood of containing objects of interest [20,23]. The rationale for integrating selective search stems from the challenges posed by mobile phone images, which often contain significant extraneous areas outside the hemocytometer grid. Selective search efficiently proposes a diverse set of candidate object locations, allowing the CNN to then classify these proposals as ROI or non-ROI with high precision. This two-step process effectively filters out irrelevant background noise and accurately pinpoints the hemocytometer area, which is crucial for subsequent cell counting. This is particularly advantageous over simpler region proposal methods in mitigating the ‘larger than intended ROI’ errors noted in our results. A representative input image is shown in Fig 3a, with the algorithmically detected candidate ROIs illustrated in Fig 3b. These candidate regions are fed into the CNN, which classifies them as ROI or non-ROI, enabling precise cropping of relevant areas for further analysis. Fig 3c and 3d provide examples of correctly and incorrectly classified regions, respectively.

The primary distinction between these two techniques lies in their operational focus: selective search isolates specific regions with potential object presence, while the CNN performs high-level classification of the entire image content. Their combination yields a robust solution for object detection and recognition in complex biological imaging scenarios [20,23].

### Cell counting

As described in the previous section, a Convolutional Neural Network (CNN) is utilized to identify and extract the region of interest (ROI) containing cells from images captured using mobile phone cameras. Following ROI extraction, a series of image processing techniques are employed to detect and enumerate the cells within the selected region. To enhance detection accuracy, multiple algorithms and filtering methods are applied, including morphological operations, edge detection, and the Hough Circle Transform. These methods work in combination to improve the precision of cell identification and minimize false detections.

### Morphological filling

Morphological image processing techniques were applied to modify the size, shape, structure, and connectivity of objects in the image through binary erosion, dilation, opening, closing, and reconstruction [24]. Erosion reduces and thins objects’ pixels in the image, while dilation enlarges and thickens them. Erosion of an image F by a structuring element S is mathematically represented as F⊖S. The structuring element S is positioned with its origin at (x,y), and the new pixel value is determined by the following equation [25]:


g(x.y)={1 if S fits F 0 otherwise  (1)

Dilation of an image F by a structuring element S is given by F⊕S, where the structuring element S is again positioned at (x,y), and the new pixel value is determined by [25]:


g(x.y)={1 if S hits F 0 otherwise  (2)

Morphological opening was employed in this process, which combines erosion and dilation. Employing this operation decreases the computational costs in image processing for cell counting by removing small objects or noise and connections in the image that lack structural integrity. The opening of an image F by a structuring element S, denoted as F∘S, is defined as erosion followed by dilation as follows [25,26]:


F∘S=(F⊖S)⊕S (3)

Prior to applying morphological operations, the cropped image is converted from RGB to grayscale. This conversion simplifies the image by reducing it to a single intensity channel, thereby enhancing the accuracy of subsequent processing steps. Morphological opening—a combination of erosion followed by dilation—is then applied to the grayscale image. This operation effectively removes small artifacts and noise while preserving the shape and structure of larger objects. The resulting image highlights the prostate cancer cells (PC3, DU-145, and LN-CaP) with enhanced contrast, while suppressing background noise, as illustrated in Fig 4b [24–27].

### Edge detection

Next, the canny edge detection algorithm is applied on the images. This algorithm processes the image and detects the edges within the image. The result is a binary image where edges are shown with white pixels and the rest of the image is covered with black pixels. In this investigation, the objects are mostly cells with almost circular edges, but some gridlines are also detected as shown in Fig 4c [27–29].

### Hough circle transform

The Hough Circle Transform (HCT) is employed to detect circular structures within the processed image, specifically targeting the shapes of individual cells while excluding non-relevant features such as grid lines. This technique is particularly effective in identifying circular contours by leveraging parameterized edge information. In this study, the transform’s sensitivity was carefully adjusted to reduce false positives, particularly near the grid lines present in hemocytometer images. By fine-tuning parameters such as edge thresholds and the expected range of radii, the accuracy of circle detection—and consequently, cell enumeration—was significantly enhanced.

The Hough Circle Transform operates on the principle of detecting circular shapes based on the equation of a circle $r^{\text{2}}={\left(x-a\right)}^{\text{2}}+{\left(y-b\right)}^{\text{2}}$ [30]. For effective circle detection, several key parameters are utilized:

- **Param1**: This parameter represents the threshold for edge detection, essentially controlling the brightness threshold or edge strength necessary for identifying potential circles in the image.
- **Param2**: This parameter determines the threshold for circle smoothness, which is influenced by the number of points on the circle. Increasing this value typically results in a higher number of detected circles, as it lowers the sensvity for the circle’s smoothness criteria.
- **MinRadius**: The minRadius parameter defines the minimum radius of circles that the algorithm should detect, allowing control over the scale of detected features.
- **MaxRadius**: The maxRadius parameter sets the maximum allowable radius for detected circles, further refining the detection process to target specific circular features.

By carefully tuning these parameters, the accuracy of cell detection was optimized, minimizing the incidence of false positives and ensuring alignment with the true cellular structures in the image. The final step in the process involved implementing a conditional statement to verify the perimeter of each detected circle, allowing for the enumeration of individual cells. The output of this automated cell counting procedure is illustrated in Fig 4d [30–32].

To enhance user accessibility and streamline the cell counting process, a web-based platform was developed for automated cell quantification. On the homepage, users can select from various cell types—including white blood cells (WBC), red blood cells (RBC), PC3, LN-CaP, and DU-145 prostate cancer cell lines (Fig 5a). After selecting the desired cell type, the platform directs users to an upload interface where images corresponding to each chamber can be submitted (Fig 5b and 5c). Upon uploading the images and entering the appropriate dilution factor, the system processes the data and displays the final cell count on the screen (Fig 5d).

## Results

The proposed convolutional neural network (CNN) model, in conjunction with the implemented image processing techniques, demonstrated strong performance in the automatic detection and enumeration of prostate cancer cell lines PC3, LN-CaP, and DU145. This section presents a comprehensive evaluation of the system’s performance, including metrics from the training and validation phases, as well as the accuracy of the final cell counting results.

### Performance evaluation across key metrics

The performance evaluation of the CNN model involves analyzing key metrics including recall, precision, and F1-score. These indicators are essential for assessing the model’s effectiveness in identifying regions of interest (ROIs) and accurately extracting them from images. Accuracy represents the overall correctness of the model, defined as the proportion of true results (both true positives and true negatives) relative to the total number of cases. However, in cases of class imbalance—such as when non-ROI regions significantly outnumber ROI regions—accuracy alone may not be a reliable measure of performance. Accuracy is calculated as follows:


$$Accuracy=\frac{TP+TN}{TP+TN+FP+FN}$$ (4)

where, TP, TN, FP, and FN are true positive, true negative, false positive, and false negative, respectively. Recall, also known as sensitivity, measures the model’s ability to correctly identify all relevant ROIs. A high recall indicates that the model successfully captures most of the ROIs, minimizing the number of false negatives.


$$Recall=\frac{TP}{TP+FN}$$ (5)

Precision evaluates the accuracy of the model in identifying positive samples, i.e., how many of the predicted ROIs were actually correct. High precision ensures that the identified ROIs are indeed relevant, minimizing the occurrence of false positives.


$$Precision=\frac{TP}{TP+FP}$$ (6)

The F1 score integrates precision and recall into a single metric to gain a better understanding of model performance. It is particularly useful when there is an uneven class distribution, as it gives a more comprehensive index for the model’s performance.


$$F\text{1}\;score=\text{2}\times \frac{Precision\;\times Recall}{Precision+Recall}$$ (7)

Macro Average Recall is the average recall across all the classes, ROI and non-ROI, treating each class equally regardless of its size.


$$Macro\;Average\;Recall=\frac{\sum_{i=\text{1}}^{n}{Recall}_{i}}{n}$$ (8)

Macro Average Precision is the average precision across all the classes. It ensures that the model’s precision is consistent across different classes, highlighting its capability to correctly identify ROIs.


$$MacroAverage\;Precision=\frac{\sum_{i=\text{1}}^{n}{Precision}_{i}}{n}$$ (9)

Weighted Average Recall accounts for the number of true instances in each class (support) when calculating the average recall. This ensures that the performance of the model is weighted according to the class distribution.


$$Weighted\;Average\;Recall=\frac{\sum_{i=\text{1}}^{n}({Recall}_{i\;}\;\times \;{Support}_{i})}{\sum_{i=\text{1}}^{n}{Support}_{i}}$$ (10)

Weighted Average Precision similarly accounts for the class distribution, providing an overall precision score that reflects the importance of each class in the dataset. It is particularly useful when dealing with imbalanced datasets, as it gives a more representative evaluation of the model’s precision.


$$Weighted\;Average\;Precision\;=\;\frac{\sum_{i=\text{1}}^{n}({Precision}_{i\;}\;\times \;{Support}_{i})}{\sum_{i=\text{1}}^{n}{Support}_{i}}$$ (11)

The performance metrics are summarized in Table 1. The results underscore the CNN model’s robustness in processing complex visual data from input images. Precision for non-ROI regions reached 100%, indicating that the model correctly identified all areas that should not contribute to the cell count. Recall for ROI regions was also 100%, reflecting the model’s ability to detect all relevant areas for counting. However, the precision for ROI regions was 88%, suggesting the presence of some false positives, likely due to larger ROI areas that may have led to overestimated cell counts. The F1-scores for ROI and non-ROI regions were 94% and 98%, respectively. These high F1-scores indicate the model’s strong overall effectiveness, as they reflect a balanced consideration of both precision and recall.

**Table 1:** Performance indices for the CNN model.

Metric	Precision	Recall	F1-score	Support
**NON-ROI**	1	0.95	0.98	43
**ROI**	0.88	1	0.94	15
**Accuracy**	–	–	0.97	58
**Macro avg**	0.94	0.98	0.96	58
**Weighted avg**	0.97	0.97	0.97	58

**Fig 6:**
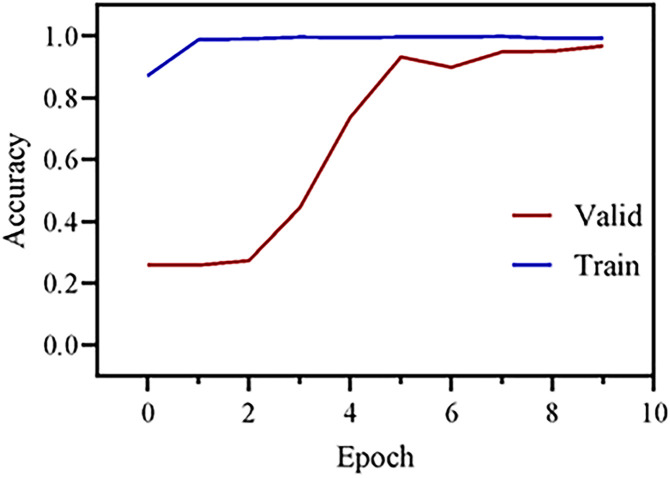
Model accuracy across epochs.

Fig 6 illustrates the training and validation accuracy across 10 epochs, highlighting the model’s learning trajectory. During training, the CNN model exhibited rapid learning, as indicated by a sharp increase in training accuracy, which stabilized at approximately 97% after only a few epochs. The validation accuracy similarly increased, rising from 27% to 95%, eventually approaching the training accuracy. This convergence is a positive indicator of the model’s generalization capability, suggesting that it successfully avoided overfitting—a common challenge in deep learning. The narrow gap between the training and validation accuracy curves further emphasizes the model’s robustness, confirming its consistent performance across both training and validation datasets. Although a slight early divergence between the two curves was observed, it suggests that initial overfitting was effectively mitigated through regularization techniques and continued training. The eventual alignment of the curves demonstrates that the model overcame early overfitting, maintaining strong performance across various data subsets.

### Cell counting accuracy

The primary objective of the CNN model was to accurately extract and forward the ROI image to the image processing algorithm. Subsequently, the image processing algorithm detects and counts the cells, presenting the results to the operator. The model was evaluated for each of the targeted cell lines. Its performance was compared with manual cell counting, a traditional method commonly used in laboratory settings. The accuracy of the system was assessed using the following equation:


$$Accuracy\;=\frac{Measured\;Blood\;cell\;Count}{\;Actual\;Blood\;cell\;count}\;\times \text{1}\text{0}\text{0}$$ (12)

**Fig 7:**
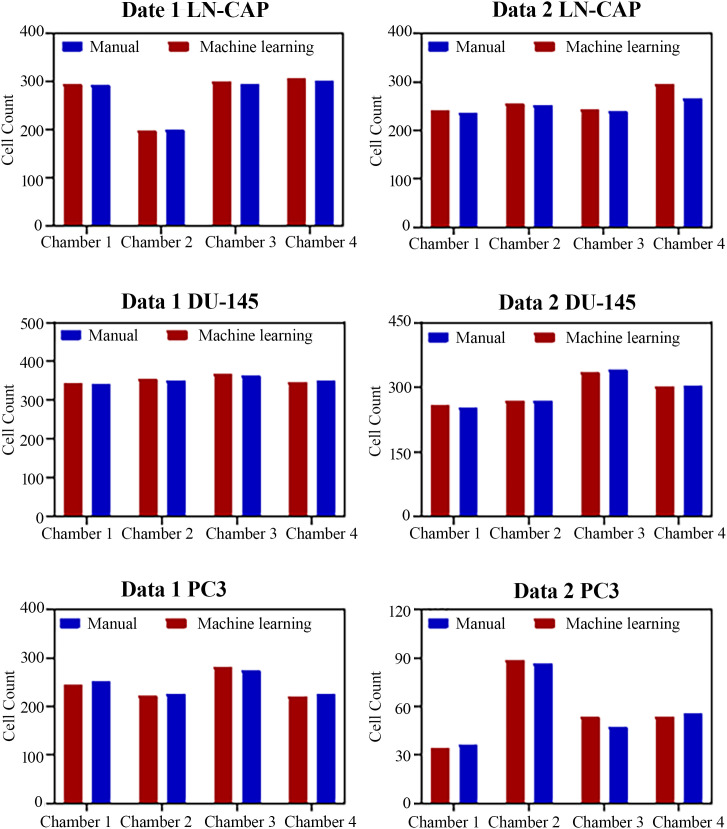
Number of manual and automated counted cells for each cell category.

The average accuracy of the image processing system was determined by calculating the mean accuracy across all evaluated datasets, yielding an overall value of 98.4%. This accuracy was derived from comparing the automated cell counts from our system with ground truth manual counts provided by an experienced expert. For each cell line (PC3, LN-CaP, DU-145), two distinct datasets were used for evaluation. Specifically, for each dataset (Data 1 and Data 2), the system processed four images (chambers) per cell line, as demonstrated in our `cell_counting` function which iterates `img_number in range (0,4)`. The total cell count for each cell line and dataset represents the sum of counts from these four chambers. The error percentages presented in Table 2 are computed on a per-dataset basis, reflecting the overall discrepancy between automated and manual counts for that specific dataset and cell type. For instance, in Data 1, manual cell counts typically ranged from approximately 200–350 cells per chamber, leading to total counts between 800–1400 cells per cell line evaluation (e.g., as depicted in Fig 7). The primary source of error in the image processing pipeline was attributed to inaccuracies in the CNN model, specifically when the extracted ROIs were larger than intended. Table 2 presents the cell counting error percentage for two representative datasets, showing that the model’s error across all cell types remained minimal. Slightly higher error rates were observed in certain sets—particularly those involving LN-CaP cells—due to factors such as variations in image quality, inconsistencies in ROI dimensions, and the presence of overlapping cells, all of which can contribute to discrepancies. Recognizing and addressing these limitations is essential for further improving model performance. Despite these challenges, the relatively low overall error highlights the system’s strong potential for both clinical and laboratory use. Fig 7 compares manual and automated cell counts across four chambers for each cell line, showing that the error in all cases remained below 4.1%.

**Table 2:** Cell counting error percentage across different sets.

Cell	Error	
	Data 1	Data 2
**PC3**	0.8%	1.7%
**LN-CAP**	1.1%	4.1%
**DU-145**	0.5%	0.2%

**Fig 8:**
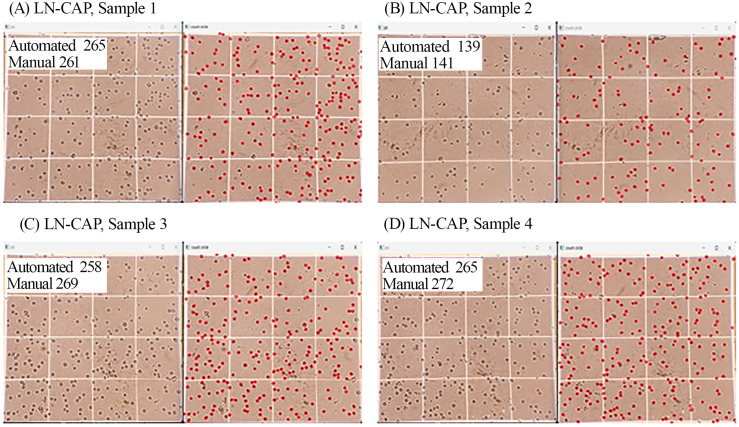
Four different cell counting samples of LN-CAP.

Fig 8 displays the automated cell counting results for LN-CaP cells, with red circles indicating the detected cells. As previously discussed, the image processing algorithm failed to identify some cells located near the borders of the image, primarily due to inaccuracies in the ROI detection stage involving the CNN model and the selective search algorithm. These errors in ROI localization resulted in incorrect image boundaries, subsequently leading to inaccuracies in the final cell count.

## Conclusion

This study presents a robust and accessible system for the automated counting of prostate cancer cells, integrating deep learning and image processing techniques. By employing a CNN for accurate region of interest (ROI) detection and applying advanced image processing methods—including morphological operations, edge detection, and Hough Circle Transform—the system effectively detects and enumerates PC3, LN-CaP, and DU145 cells. Designed to process images captured via mobile phones, the platform offers a user-friendly and cost-efficient solution suitable for both clinical and laboratory environments. The system demonstrated high accuracy, with an average performance of 98.4%, and minimal deviation from manual counting. However, certain limitations were observed, particularly in cases involving inaccurate ROI extraction due to image quality issues or overlapping cells. Addressing these challenges through further model refinement will enhance the system’s reliability. Overall, the proposed method provides a promising tool for improving the efficiency, scalability, and consistency of cell counting in prostate cancer research and diagnostics. Looking forward, we are actively exploring partnerships for external user testing and validation of the web-based platform in diverse laboratory and clinical settings. Furthermore, there are plans to open-source the platform and make it publicly available, fostering broader research, validation, and collaborative development within the scientific community.
